# On the selection of thresholds for predicting species occurrence with presence‐only data

**DOI:** 10.1002/ece3.1878

**Published:** 2015-12-29

**Authors:** Canran Liu, Graeme Newell, Matt White

**Affiliations:** ^1^ Arthur Rylah Institute for Environmental ResearchDepartment of Environment, Land, Water and PlanningHeidelbergVictoria3084Australia

**Keywords:** *F* measure, presence‐only, prevalence, species distribution modeling, specificity, threshold

## Abstract

Presence‐only data present challenges for selecting thresholds to transform species distribution modeling results into binary outputs. In this article, we compare two recently published threshold selection methods (maxSSS and max*F*_pb_) and examine the effectiveness of the threshold‐based prevalence estimation approach. Six virtual species with varying prevalence were simulated within a real landscape in southeastern Australia. Presence‐only models were built with DOMAIN, generalized linear model, Maxent, and Random Forest. Thresholds were selected with two methods maxSSS and max*F*_pb_ with four presence‐only datasets with different ratios of the number of known presences to the number of random points (KP–RP_ratio_). Sensitivity, specificity, true skill statistic, and *F* measure were used to evaluate the performance of the results. Species prevalence was estimated as the ratio of the number of predicted presences to the total number of points in the evaluation dataset. Thresholds selected with max*F*_pb_ varied as the KP–RP_ratio_ of the threshold selection datasets changed. Datasets with the KP–RP_ratio_ around 1 generally produced better results than scores distant from 1. Results produced by We conclude that maxF_pb_ had specificity too low for very common species using Random Forest and Maxent models. In contrast, maxSSS produced consistent results whichever dataset was used. The estimation of prevalence was almost always biased, and the bias was very large for DOMAIN and Random Forest predictions. We conclude that max*F*_pb_ is affected by the KP–RP_ratio_ of the threshold selection datasets, but maxSSS is almost unaffected by this ratio. Unbiased estimations of prevalence are difficult to be determined using the threshold‐based approach.

## Introduction

Species distribution modeling has become an important tool to tackle issues in ecology, evolutionary biology, biogeography, and conservation planning. Species distribution data are commonly obtained from herbaria, natural history museums, and government databases to develop these models. These data sources generally only provide presence data for species and rarely provide information on absences. Many modeling techniques have been introduced or adapted to deal with these types of data. Some techniques use presence data exclusively, for example, BIOCLIM (Busby 1991), DOMAIN (Carpenter et al. 1993), LIVES (Li and Hilbert 2008), and one‐class SVM (Guo et al. 2005; Drake et al. 2006), while other techniques use both presence data and accompanying pseudo‐absence data (often random points) that represent the background context for the model. This latter group of modeling approaches include GARP (Stockwell and Peters 1999), ENFA (Hirzel et al. 2002), Maxent (Phillips et al. 2006), and all the group‐discrimination techniques including ANN (Pearson et al. 2002), generalized linear model (GLM), GAM, boosted regression tree (Elith et al. 2006), Random Forest (Cutler et al. 2007), and two‐class SVM (Guo et al. 2005). Point process modeling has also been introduced to build models with presence‐only data (Warton and Shepherd 2010; Chakraborty et al. 2011; Aarts et al. 2012). Most of these techniques produce continuous predictions, which may be considered as relative suitabilities for species occurrence.

Applied ecological problems such as climate change impacts (Bush et al. 2014; Muir et al. 2015), invasive species impacts (Buckland et al. 2014), reintroduction sites identification (Bleyhl et al. 2015), and conservation planning (Abade et al. 2014) often require binary models of distributions, and a threshold is needed to transform the continuous results into a binary product. Although this discretization may lose some information (Guillera‐Arroita et al. 2015), it is still the only way for some problems, especially those involving species range estimation (Syfert et al. 2014; Saupe et al. 2015). Although there are many threshold selection methods for presence/absence data (Fielding and Bell 1997; Liu et al. 2005; Jiménez‐Valverde and Lobo 2007; Freeman and Moisen 2008; Nenzén and Araújo 2011), there are very few methods proposed for use with presence‐only data (Phillips et al. 2006; Pearson et al. 2007; Li and Guo 2013; Liu et al. 2013a). The minimum predicted value for the training sites has been used as threshold (Phillips et al. 2006), termed “lowest presence threshold” (Pearson et al. 2007). With this method, sensitivity achieves its maximum value 1 with the evaluation data, but it is extremely sensitive to low sample sizes (Bean et al. 2012), and sometimes it predicts species' presence everywhere in the study area (Phillips et al. 2006). More generally, different levels of sensitivity can be set so that different thresholds can be obtained (e.g., sensitivity = 0.9) (Pearson et al. 2004). However, as this approach only considers sensitivity, its usefulness remains limited.

Maximizing the sum of sensitivity and specificity (maxSSS), one of the best threshold selection method for presence/absence data (Liu et al. 2005), has been proved valid to use with presence‐only data when random points are used instead of true absences, where sensitivity is the proportion of correctly predicted presences among all the presences (which is also called recall in other fields), and specificity is the proportion of correctly predicted absences among all the absences (Liu et al. 2013a). Three criteria were proposed (i.e., objectivity, equality and discriminability) as sound principles for threshold selection. Specifically, the threshold should be objectively selected, and the selected threshold should be identical irrespective of either presence/absence data or presence‐only data being used in the selection, at least for large samples. Additionally, discrimination between presence and absence rather than between presence and random point should be optimized. maxSSS satisfies all the three criteria.

Li and Guo (2013) derived a statistic *F*_pb_ (which will be defined in the next section) based upon the presence‐background (which is defined as random points) data acting as a surrogate for the *F* measure (i.e., the harmonic mean of positive predicted value and sensitivity), and used it to select thresholds, where positive predicted value (also called precision) is the proportion of correctly predicted presences among all the predicted presences. They found that thresholds selected by maximizing *F*_pb_ (i.e., max*F*_pb_) and by maximizing *F* (i.e., max*F*) were similar. However, in calculating *F*, only correctly predicted presences, observed presences, and predicted presences are used, and the total number of evaluation data points are not used in the calculation, although this value is known. This makes true absences unconstrained in all implementations of max*F*, which may result in very low specificity. Consequently, the behaviors of both max*F* and max*F*_pb_ require further examination.

These authors also proposed estimating species prevalence from the transformed binary predictions with max*F*_pb_ and found that the estimated prevalence was very accurate with root‐mean‐square error (RMSE) < 0.081 for all the models (here for prevalence estimation RMSE is always between 0 and 1, and the smaller the RMSE, the better the estimation). This means that the mean absolute bias of the estimation is also <0.081, since arithmetic mean is less than quadratic mean (Pachpatte 2005). Because of the potential problems with max*F* as mentioned above, the effectiveness of this threshold‐based prevalence estimation approach also needs to be further examined.

We use a simulation approach in this article to compare the two threshold selection methods max*F*_pb_ and maxSSS to examine the difference in their performance under different combinations of modeling techniques and species with varying prevalence. We focus on their response to threshold selection datasets with different ratios of the number of known presences to the number of random points (KP–RP_ratio_) and also investigate the performance of the threshold‐based prevalence estimation approach when models are trained with different number of presences.

## Methods

### Threshold selection methods

For presence/absence data, the identities of both presence and absence data are known, and the related confusion matrix and calculation of various accuracy metrics can be found in many papers (Liu et al. 2011). For presence‐only data, we used random points (or more generally pseudo‐absences) as the “absence” component of the evaluation data in addition to the presence component (i.e., known presences), and the related confusion matrix can be constructed similarly. Among the four cells of this matrix, true presences and false absences are calculated in the same way as with presence/absence data, and the “true absences” and “false presences” are calculated with pseudo‐absences, which are the predicted absences (i.e., with predicted values below the threshold) among all the pseudo‐absences, and the predicted presences (i.e., with predicted values above the threshold) among all the pseudo‐absences.

For a metric ***M*** (calculated with presence/absence data), its presence‐only counterpart is expressed as *M*′ (calculated with presence/pseudo‐absence data, i.e., the apostrophe (′) indicates a value based on presence‐only data). Liu et al. (2013a) derived that

$${\text{SSS}}^{\prime }={\text{Se}}^{\prime }+{\text{Sp}}^{\prime }=s+\left(1-s\right)\left(\text{Se}+\text{Sp}\right)=s+\left(1-s\right)\text{SSS},$$

and therefore,

$${\text{TSS}}^{\prime }=\left(1-s\right)\text{TSS},$$

where SSS is the sum of sensitivity (Se) and specificity (Sp), TSS = Se + Sp – 1 = SSS – 1 is the true skill statistic, *s* is the proportion of true presences among the pseudo‐absences, and it is therefore the species prevalence if random points are taken as pseudo‐absences (Liu et al. 2013a). Because *s* is a constant (between 0 and 1) for a dataset, SSS′ and TSS′ are monotonically increasing functions of SSS and TSS, respectively. If a threshold makes any of the four indices (i.e., SSS′, TSS′, SSS, and TSS) reach its maximum, then it will make the other three reach their maximum. Therefore, maximizing the four indices is equivalent in terms of selecting thresholds. Theoretically, this is not affected using different sets of data even with different values of the constant *s*. Suppose we have two datasets each containing a presence component and a pseudo‐absence component, the ratios of the number of true presences among the pseudo‐absences to the number of pseudo‐absences for the two datasets are *s*_1_ and *s*_2_, respectively. According to the above discuss‐ion, we have ${\text{SSS}}_{1}^{\prime }=s_{1}+\left(1-s_{1}\right)\text{SSS}$ and ${\text{SSS}}_{2}^{\prime }=s_{2}+\left(1-s_{2}\right)\text{SSS}$. Since both *s*_1_ and *s*_2_ are constants, SSS is always maximized by maximizing either SSS_1_ or SSS_2_. Therefore, maxSSS is not affected by different datasets. For simplicity, in the following we will not explicitly write the apostrophe. From these discussions and also considering TSS is a well‐recognized accuracy measure, maxSSS satisfies all the three criteria.

$F_{\mathrm{pb}}=\left(2p^{\prime \prime }r^{\prime }\right)/\left(p^{\prime \prime }+r^{\prime }\right)$ is the harmonic mean of *r*′ and *p*″, which was proposed to be a surrogate of the accuracy measure *F* = 2*pr*/(*p* + *r*), where *r* and *r*′ are the sensitivities (i.e., recall) of the model calculated with presence/absence data and presence‐only data respectively, *p* is the positive predicted value (i.e., precision), and *p*″ is defi‐ned as the ratio of true presences (i.e., correctly predicted presences among all the known presences in the dataset) to the (pseudo) false presences (i.e., predicted presences among all the random points in the dataset) (Li and Guo 2013). The proposed threshold selection method was based on maximizing *F*_pb_ (hereafter max*F*_pb_). It can be derived that $p=p^{\prime \prime }/c$ (Li and Guo 2013), where $c=n_{1}/\left(\pi n_{0}\right)$, *n*_1_ is the number of randomly sampled presences, *n*_0_ is the number of random points, and *π* is the species prevalence. Since the calculation of *r* and *r*′ only uses true presences, they should be equal for large samples, that is, *r* = *r*′. Therefore, we have *F*_pb_ = 2*pr*/(*p* + *r*/*c*), which is a function of *p* and *r* with a parameter *c*. However, *c* is unknown prior to the analysis, and it will vary each time we alter the KP–RP_ratio_. Different values of c can be obtained in a simple situation where we use the same set of presences but varying number of random points.

When *c*→0, that is, when there are much fewer known presences than the true presences within the random points (which can be achieved by taking a large number of random points), *F*_pb_→0; when *c*→1, that is, there are as many known presences as the true presences in the random points, $F_{\mathrm{pb}}\to 2pr/\left(p+r\right)=F$; when *c*→∞, that is, there are much more known presences than the true presences in the random points, *F*_pb_→2r. It is therefore unclear what we are really measuring each time we apply this metric to a dataset since we do not know how many presences there are in the random points. We must emphasize that the two components (presences and pseudo‐absences) come from two separate sampling processes in this presence‐only situation, and random sampling can only be (ideally) assumed for each component, and their combination cannot be assumed from simple random sampling any more. Therefore, theoretically c can take any positive value.

Although the two extreme situations (*c* →0 and *c*→∞) are unlikely to occur in real situations, the above reasoning illustrates that varying values of *c* results in different accuracy measures. This means that if we use datasets with different KP–RP_ratios_ for the same model, we would subsequently have different criteria even with the same name *F*_pb_. Therefore, if we select thresholds by max*F*_pb_, it is highly likely that different thresholds will be selected, even with two large datasets.

### Data creation and analysis

We developed six virtual species distributed within a 250 × 250 km area in central–western Victoria, Australia. Eighteen environmental variables were available included relevant bioclimatic, topographical, and radiometric variables (Liu et al. 2013b). We used principal component analysis to extract three principal components accounting for more than 65% of the total variation. Three normalized environmental variables (*x*_j_, *j* = 1, 2, 3) were obtained by subtracting the mean from each component and being divided by its standard deviation. All of the environmental data were resolved to 1 × 1 km, creating a total of *n*_T_ = 62,500 cells.

In this article, we adopted the probabilistic approach to simulating species distributions (Meynard and Kaplan 2013). The probability of the species occurrence was calculated for each of the six species at site *i* (*i* = 1, 2,…,*n*_*T*_) with environmental data *X*_*i*_ = (*x*_*i*1_, *x*_*i*2_, *x*_*i*3_), using

$$p_{i}=\frac{1}{1+e^{-f(X_{i})}},$$

where *f*(*X*_*i*_) = *a*_0_ + a_1_ *x*_*i*1_ + *a*_2_ *x*_*i*2_ + *a*_3_ *x*_*i*3._ The parameters *a*_*j*_ (*j* = 0, 1, 2, 3) for the six species are shown in Appendix S1.

In order to form a binary distribution (i.e., realization) for each species, for each site *i* (*i* = 1, 2,…,*n*_T_), we carried out a Bernoulli trial with probability *p*_*i*_. If the outcome was 1, site *i* was labeled presence; otherwise, it was labeled absence. A set of realizations for the six virtual species are shown in Appendix S2. This procedure was applied for 100 realizations for each species. It can be seen that the above procedure is just the inverse process of logistic regression. While logistic regression is used to estimate the coefficients of the model with both explanatory and response variables, here we calculated the values of the response variable with known coefficients of the model and explanatory variables.

Four modeling techniques (DOMAIN, GLM, Maxent, and Random Forest) were used. All calculations were carried out in R 2.15.2 (R Development Core Team, 2012). The R packages stats 2.15.2, randomForest 4.6‐10, and dismo 0.8‐11 together with Phillips & Dudík's program (Phillips and Dudík 2008) were used to implement GLM, Random Forest, and Maxent, respectively. DOMAIN (Carpenter et al. 1993) was implemented with our custom programming of the algorithm. For each species, we randomly selected 50 presences and 5000 random points as model training data, but using so many random points in the training data resulted in Random Forest models with very low accuracy. Consequently, we used a ratio of 2:1 for the number of random points compared to the number of presences for Random Forest models, that is, 100 random points. This strategy was adopted by Liu et al. (2013a, 2013b), and similar strategy has already been recommended by others (Barbet‐Massin et al. 2012). Higher accuracies can be achieved using ensembles of many such models (Barbet‐Massin et al. 2012); however, this is unnecessary for this article. Since the 50 presences were randomly sampled from the entire study area, they were representative of all the presences, and therefore, generally high‐quality models could be built with them. Unlike using a large number of presences from which models with high accuracies would always be obtained, using a smaller number of presences we could build models with wider range of accuracies, which is better for comparative studies as in this article.

A test dataset was created by selecting 3000 *π* presences and 3000 (1–*π*) absences from the full dataset excluding the training data, where *π* was species prevalence. Four validation datasets were created for selecting thresholds by selecting *n1val* presences from all the presences excluding the training data and test data, and *nrpval* random points from the full dataset, where *n1val *= 50 and *nrpval *= 5000, *n1val *= 50 and *nrpval *= 50, *n1val *= 5000 and *nrpval *= 5000, and *n1val *= 5000 and *nrpval *= 50 for the four datasets (termed po1, po2, po3, and po4), respectively. A presence/absence validation dataset (pa1) was also created by selecting 5000*π* presences and 5000 (1–*π*) absences from the full dataset excluding the training data and test data. This presence/absence dataset was used to select thresholds using methods max*F* and maxSSS, and the four presence‐only datasets were used to select thresholds using max*F*_*pb*_ and maxSSS′ (since the formulae are exactly the same for maxSSS and maxSSS′, we do not differentiate them further). For the latter, the (pseudo) specificity was calculated using the pseudo‐absences (i.e., the random points).

The results were evaluated against the test data. The area under the receiver operating characteristic curve (AUC) was calculated with the original model predictions to show the general accuracy of the models. The predictions after being transformed with the selected threshold were evaluated with sensitivity, specificity, *F*, and TSS (Liu et al. 2011).

In order to investigate the performance of estimating prevalence with the threshold‐based approach, the prevalence was estimated when a threshold was selected by calculating the ratio of the number of predicted presences to the total number of points in the evaluation dataset. This is equivalent to the ratio of the predicted area of presence (where model predictions were above the selected threshold) to the total study area.

## Results

The AUC values (across the 100 realizations for each of the six species with each of the four modeling techniques) ranged from 0.75 to 0.98 for DOMAIN models, from 0.80 to 1 for GLM models, from 0.66 to 0.99 for Maxent models, and from 0.61 to 0.96 for Random Forest models (Appendix S1).

**Figure 1:**
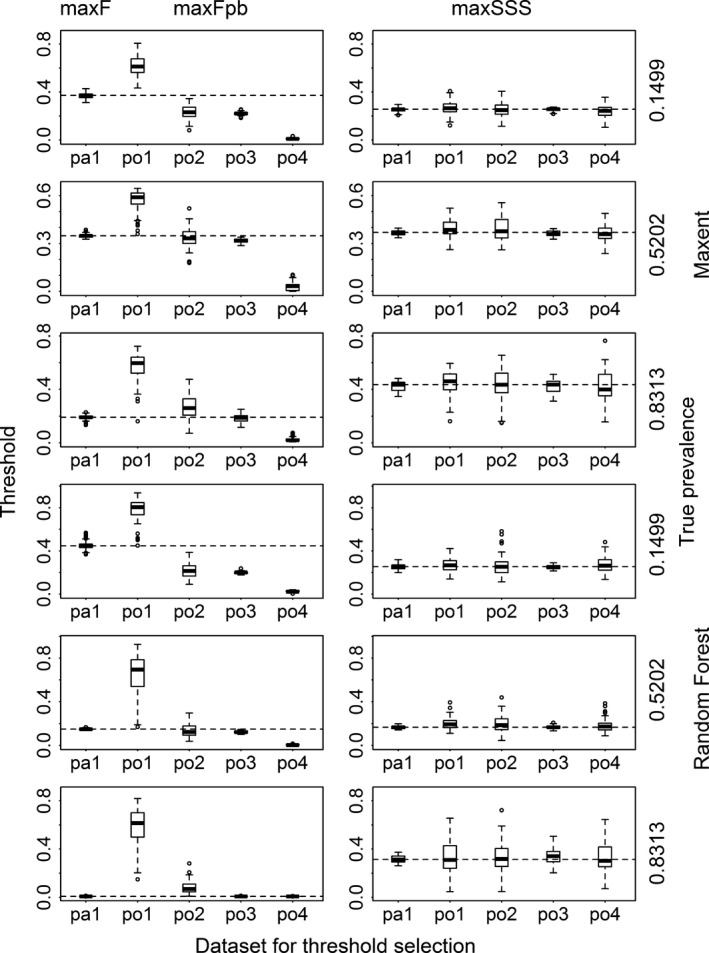
Thresholds selected using max*F* with presence/absence dataset (pa1), using max*F*_pb_ with four presence‐only datasets (po1, po2, po3, and po4), and using maxSSS with all the five datasets for Maxent and Random Forest models for three virtual species with low, intermediate, and high prevalence. The dashed lines correspond to the median thresholds selected using max*F* and maxSSS with pa1.

The four presence‐only datasets produced very different thresholds when max*F*_pb_ was used. Thresholds produced with the dataset po1 were much higher and those produced with the dataset po4 were much lower than those produced with the presence/absence dataset (pa1) using max*F* (Fig. 1 and Appendix S3). Those produced with the datasets po2 and po3 were also lower than their presence/absence counterparts for species with low prevalence, especially for Maxent and Random Forest models. This contrasts with the use of maxSSS, where the median thresholds produced with the four presence‐only datasets and the presence/absence dataset were almost the same.

**Figure 2:**
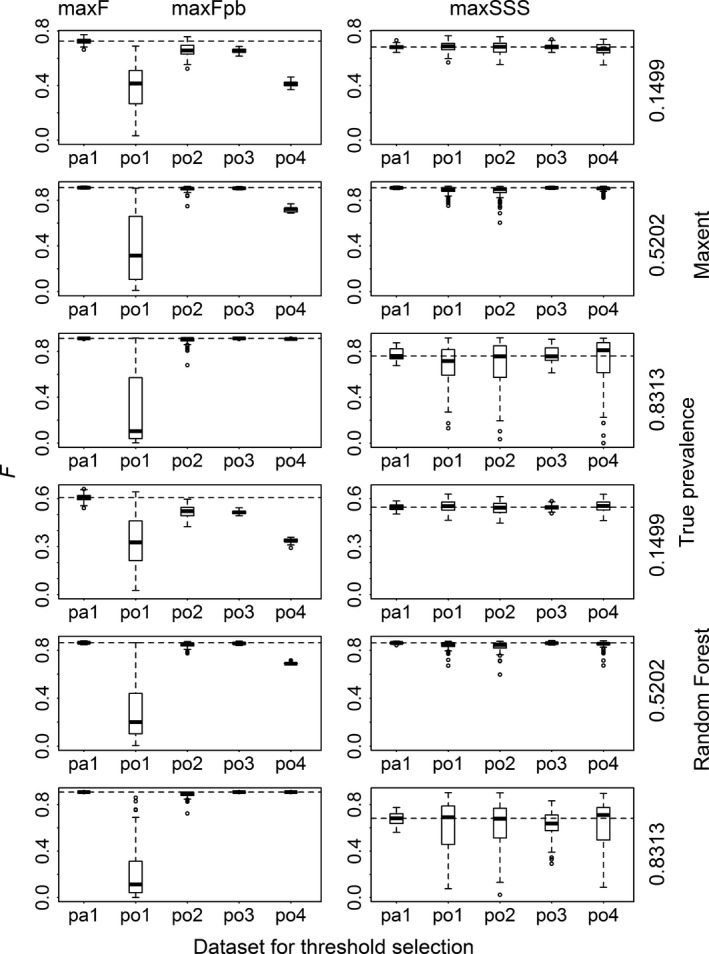
*F* measures calculated for the results transformed with the thresholds selected using max*F* with presence/absence dataset (pa1), using max*F*_pb_ with four presence‐only datasets (po1, po2, po3, and po4) and using maxSSS with all the five datasets for Maxent and Random Forest models for three virtual species with low, intermediate, and high prevalence. The dashed lines correspond to the median *F* of those using max*F* and maxSSS with pa1.

**Figure 3:**
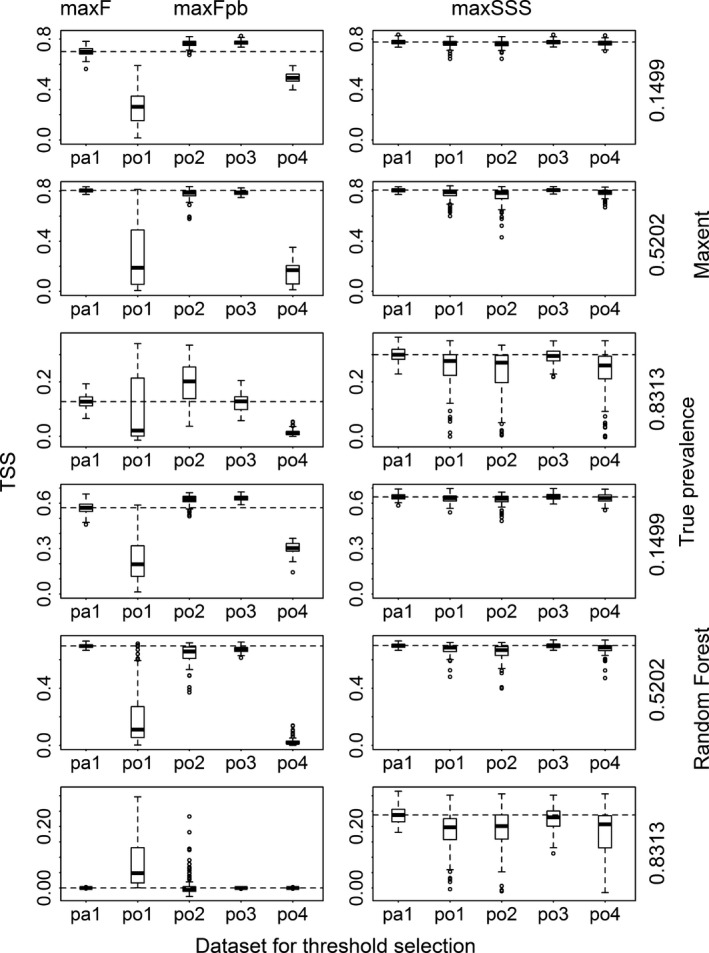
True skill statistic (TSS) calculated for the results transformed with the thresholds selected using max*F* with presence/absence dataset (pa1), using max*F*_pb_ with four presence‐only datasets (po1, po2, po3, and po4) and using maxSSS with all the five datasets for Maxent and Random Forest models for three virtual species with low, intermediate, and high prevalence. The dashed lines correspond to the median TSS of those using max*F* and maxSSS with pa1.

**Figure 4:**
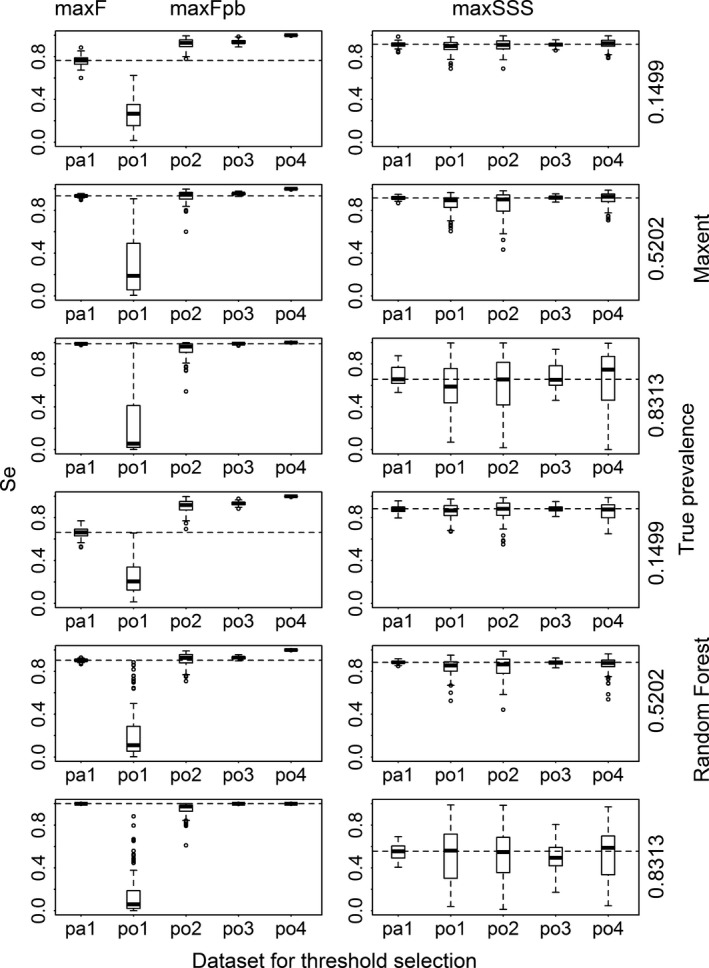
Sensitivity (Se) calculated for the results transformed with the thresholds selected using max*F* with presence/absence dataset (pa1), using max*F*_pb_ with four presence‐only datasets (po1, po2, po3, and po4) and using maxSSS with all the five datasets for Maxent and Random Forest models for three virtual species with low, intermediate, and high prevalence. The dashed lines correspond to the median sensitivity of those using max*F* and maxSSS with pa1.

**Figure 5:**
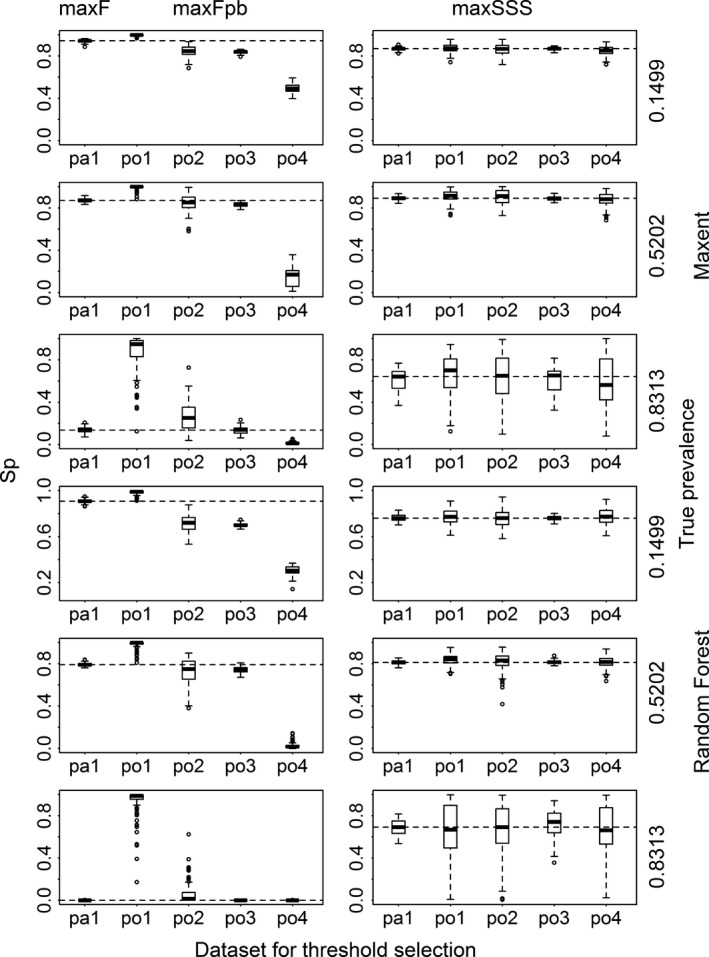
Specificity (Sp) calculated for the results transformed with the thresholds selected using max*F* with presence/absence dataset (pa1), using max*F*_pb_ with four presence‐only datasets (po1, po2, po3, and po4), and using maxSSS with all the five datasets for Maxent and Random Forest models for three virtual species with low, intermediate, and high prevalence. The dashed lines correspond to the median specificity of those using max*F* and maxSSS with pa1.

When assessing the binary results transformed with the thresholds selected by max*F* and max*F*_pb_, the *F* and TSS values corresponding to po1 and sometimes po4 were much lower than their presence/absence counterparts (i.e., those produced with maxF and pa1), and the *F* and TSS values corresponding to po2 and po3 were not much dissimilar to their presence/absence counterparts (Figs. 2 and 3 and Appendix S3). For the results transformed with the thresholds selected by maxSSS, the *F* and TSS values corresponding to the five datasets were roughly the same. They almost reached the best level of F corresponding to max*F* except for very common species modeled with Random Forest. For less common species, the results from max*F* and max*F*_pb_ with the two datasets po2 and po3 reached TSS similar to that from maxSSS. For common and very common species modeled with Random Forest and Maxent, max*F* and max*F*_pb_ produced results which had very low TSS (almost 0 for Random Forest models). When max*F*_pb_ was used, datasets po1 and po4 produced results very different from their presence/absence counterparts in terms of sensitivity and specificity. Dataset po1 produced results with very high specificity but very low sensitivity, while the results using dataset po4 produced opposite pattern. Datasets po2 and po3 produced results not greatly different from their presence/absence counterparts and maintained very high sensitivity with low specificity (even close to 0 for very common species when modeled with Random Forest and Maxent) (Figs. 4 and 5 and Appendix S3). When comparing the two methods, for species with low and intermediate levels of prevalence (except modeling with Random Forest), max*F*_pb_ (with po2 and po3), max*F*, and maxSSS produced similar level of sensitivity and specificity. However for species with high and intermediate levels of prevalence (when modeled with Random Forest), maxSSS produced much higher specificity than max*F* and max*F*_pb_ (with po2, po3, and po4).

**Figure 6:**
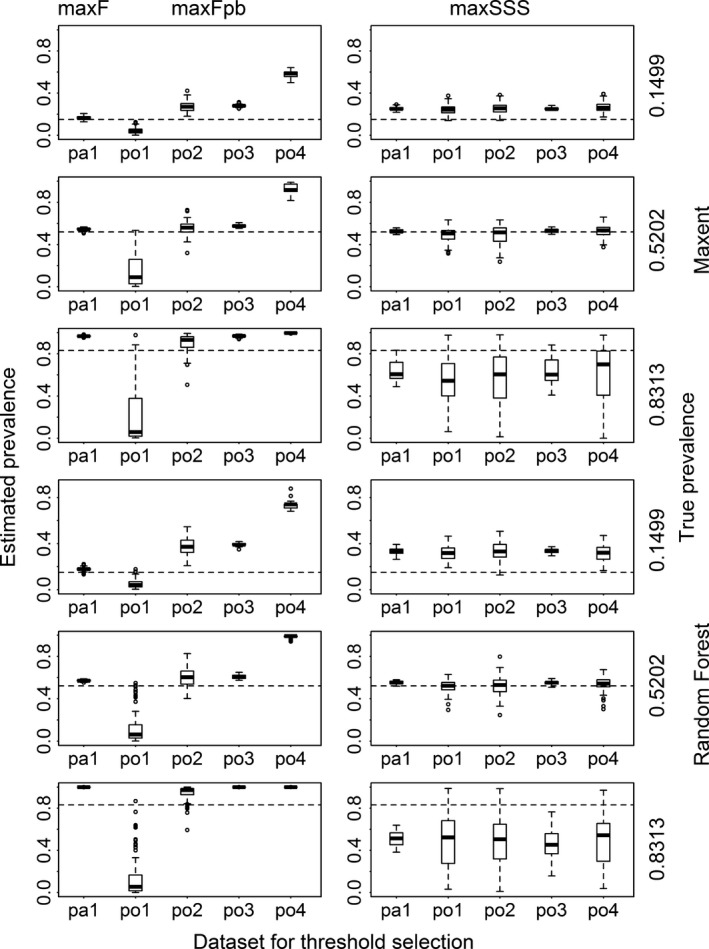
Estimated prevalence from the results transformed with the thresholds selected using max*F* with presence/absence dataset (pa1), using max*F*_pb_ with four presence‐only datasets (po1, po2, po3, and po4), and using maxSSS with all the five datasets for Maxent and Random Forest models for three virtual species with low, intermediate, and high prevalence. The dashed lines correspond to the true prevalence.

**Figure 7:**
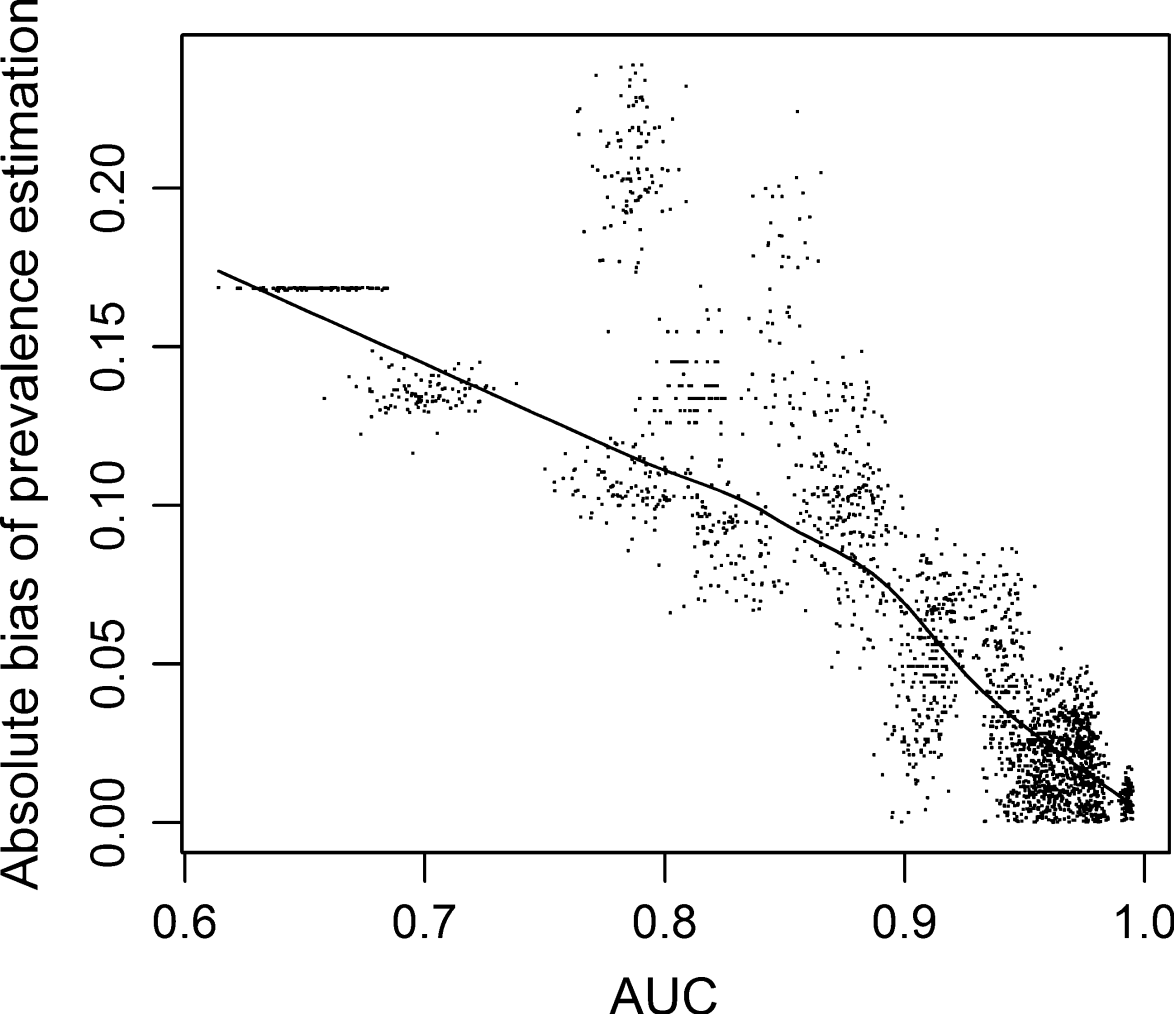
The effect of model accuracy AUC on the prevalence estimation with threshold‐based approach where the threshold was selected by max*F* with presence/absence data. Here, the absolute bias in prevalence estimation is the absolute value of the difference between estimated and true prevalence. The solid line is the smooth curve from local regression.

Prevalence was always highly underestimated by dataset po1 and overestimated by the other datasets (especially po4) when max*F*_pb_ was used (Fig. 6 and Appendix S3). It was almost always overestimated when max*F* was used, and the level of bias in the prevalence estimation by max*F* was significantly negatively correlated with the accuracy (AUC) of the models (Spearman's rank correlation coefficient = −0.8673, *P* < 0.0001, Fig. 7). When maxSSS was used, all the five datasets produced almost the same prevalence estimation in all the situations. The estimated prevalence was higher than, lower than, and similar to the true prevalence for rare species, for very common species, and for species with intermediate level of prevalence, respectively. For species with low and intermediate levels of prevalence, the prevalence estimated by max*F*_pb_ using po2 and po3 was similar to that estimated by maxSSS. But for species with high level of prevalence, there was quite large difference in the prevalence estimation between the two methods. While the former overestimated the prevalence, the latter underestimated it.

## Discussion

Our simulation results have clearly demonstrated that max*F*_pb_ selects different thresholds when using datasets with different KP–RP_ratios_. This is consistent for all the species examined and for all the modeling techniques studied. This is an undesirable property of the use of max*F*_pb_. In contrast, maxSSS always produces almost the same threshold for a model using either presence/absence data or presence‐only data, which has previously been demonstrated with an alternative simulation approach (Liu et al. 2013a).

In this study, we used datasets where the KP–RP_ratios_ were < 1 for the presence‐only dataset po1, equal to 1 for po2 and po3, and greater than 1 for po4. It may be argued that in practice it may be possible to allocate the number of random points used for threshold selection based upon the number of presences used to maintain a ratio of known presences to random points close to (or at least not far away from) 1. With this approach, it may be possible to constrain the resultant threshold to be close to that from max*F*. However, this approach depends on our prior knowledge about the prevalence of the focal species in the study area, and this is generally unknown. A further enhancement could be to apply this process iteratively by estimating several values for the prevalence and adjusting the number of random points according to the estimated prevalence. This process may potentially make the max*F*_pb_ results close to max*F* results.

However, this approach using max*F* theoretically may not be a good strategy for threshold selection. As mentioned previously, the true absences (in the confusion matrix) are totally unconstrained in the application of max*F*, and this produces results with lower sensitivity and higher specificity than those from maxSSS for rare species, and with very high sensitivity and very low specificity (even close to 0 which is much lower than that from maxSSS) for very common species. These responses are undesirable. With rare species, omission errors are much more important than commission errors, and missing suitable locations may result in poor decisions in conservation management and a contribution to further loss of biodiversity. In contrast, commission errors are more important than omission errors when considering common species, and the use of modeled outputs with high commission errors for conservation planning management may lead to poor resource allocation. In this respect, maxSSS provides better results than max*F* and max*F*_pb_ using either presence/absence data or presence‐only data.

Several authors have posited the view that prevalence cannot be determined from presence‐only data (Hastie and Fithian 2013; Lele et al. 2013; Phillips and Elith 2013; Guillera‐Arroita et al. 2015), and this is supported by our results. We have found that it is very difficult to unbiasedly estimate species prevalence with presence‐only data, even with presence/absence data using threshold‐based approach. The effectiveness of this approach strongly depends on the accuracy of the models. It seems that in order to obtain a reasonable prevalence estimation (e.g., with absolute bias < 0.1), the AUC of the models should be at least 0.9 (Fig. 7). This is difficult to achieve in practice. Relative bias <10% may be a better criterion. It is much stricter, and much smaller absolute bias is required. Therefore, it is even more difficult to achieve in practice. A large comparative study has shown that for the 226 species with various modeling techniques, only a small proportion of models reached this level of accuracy, even the most powerful modeling techniques obtained mean AUCs < 0.73 (Elith et al. 2006). Although many studies reported highly accurate models, their accuracies are often inflated because their evaluation data and model training data are usually not independent (Araujo and Guisan 2006; Veloz 2009).

In conclusion, the threshold selection method max*F*_pb_ is affected by the KP–RP_ratio_ used in the threshold selection dataset, but this is not the case for the threshold selection method maxSSS, which produces similar results when using either presence/absence or presence‐only datasets. A further disadvantage of max*F*_pb_ against maxSSS is that it produces results with lower (sometimes close to 0) specificity for very common species, impairing the utility of this method compared to maxSSS. Additionally, the effectiveness of prevalence estimation with threshold‐based approach strongly depends on the accuracy of models. Reasonable prevalence estimation requires highly accurate models, which is generally very difficult to achieve in practise.

## Conflict of Interest

None declared.

## Supporting information


**Appendix S1.** Coefficients used in the simulation for the six virtual species and the accuracy of models.
**Appendix S2.** A set of realizations of the six virtual species used in this study.
**Appendix S3.** More results on threshold selection.
