# Supplementary material for: On the selection of thresholds for predicting species occurrence with presence‐only data
Source: Ecol Evol. 2015 Dec 29;6(1):337–48. doi: 10.1002/ece3.1878 (PMC4716501; doi:10.1002/ece3.1878)
Supplement: Supplementary file 1 — Appendix S1. Coefficients used in the simulation for the six virtual species and the accuracy of models. Appendix S2. A set of realizations of the six virtual species used in this study. Appendix S3. More results on threshold selection. [file ECE3-6-337-s001.docx]

*Ecology and Evolution*

**SUPPORTING INFORMATION**

**On the selection of thresholds for predicting species occurrence with presence-only data**

Canran Liu, Graeme Newell and Matt White

**Appendix S1** Coefficients used in the simulation of the distributions for the six virtual species, their prevalence, and the accuracy (AUC: median, minimum and maximum) of the four models (DOMAIN, GLM, Maxent and Random Forest) for each species calculated across the 100 replications.

Species Coefficients Prevalence AUC

a_0_ a_1_ a_2_ a_3_ DOMAIN GLM Maxent RF

1 -8.8426 -6.9788 7.7092 -5.6771 0.1499 0.87 0.96 0.96 0.91

0.85 0.95 0.95 0.89

0.90 0.98 0.97 0.92

2 -5.7389 -6.9767 -3.5599 8.2569 0.2927 0.86 0.96 0.96 0.94

0.84 0.95 0.93 0.93

0.89 0.98 0.98 0.96

3 -4.7259 -8.9049 4.4165 -7.0258 0.4274 0.97 0.99 0.98 0.95

0.96 0.99 0.97 0.94

0.98 1.00 0.99 0.96

4 0.9924 -3.8011 5.1823 9.1061 0.5202 0.79 0.98 0.97 0.91

0.76 0.97 0.96 0.89

0.81 0.98 0.98 0.93

5 2.8220 -8.5731 4.2235 0.6703 0.7179 0.88 0.94 0.92 0.81

0.86 0.93 0.90 0.78

0.90 0.95 0.94 0.84

6 2.2955 -1.7374 2.6376 -1.3517 0.8313 0.78 0.83 0.70 0.65

0.75 0.80 0.66 0.61

0.82 0.87 0.74 0.68

**Appendix S2** A set of realisations of the six virtual species used in this study


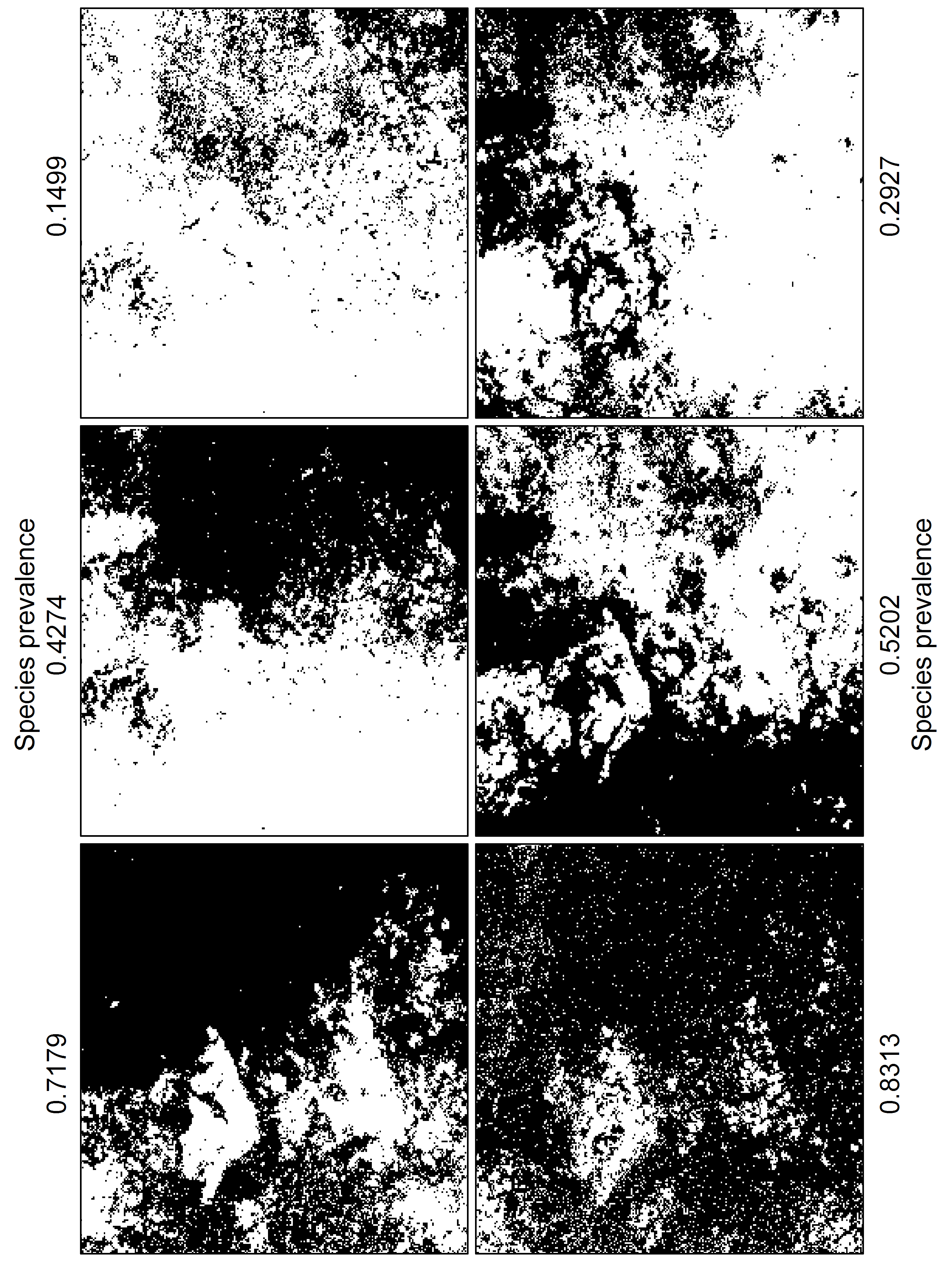


**Appendix S3** Other modelling results on threshold selection


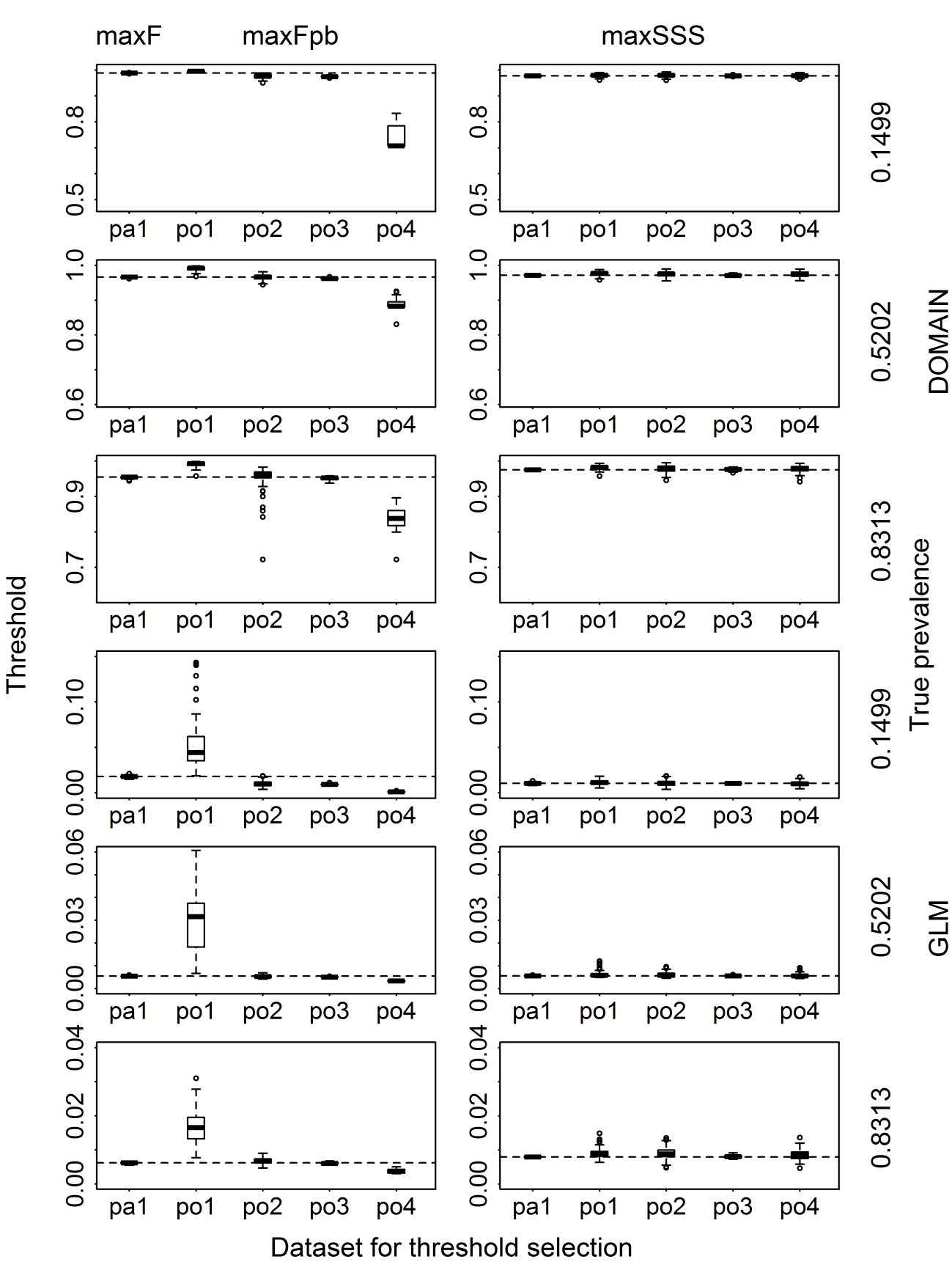


Fig. S3-1 Thresholds selected using maxF with presence/absence dataset (pa1), using maxF_pb_ with four presence-only datasets (po1, po2, po3 and po4) and using maxSSS with all the five datasets for DOMAIN and GLM models for three virtual species with three levels of prevalence (0.1499, 0.5202 and 0.8313). The dashed lines correspond to the median thresholds selected using maxF and maxSSS with pa1.


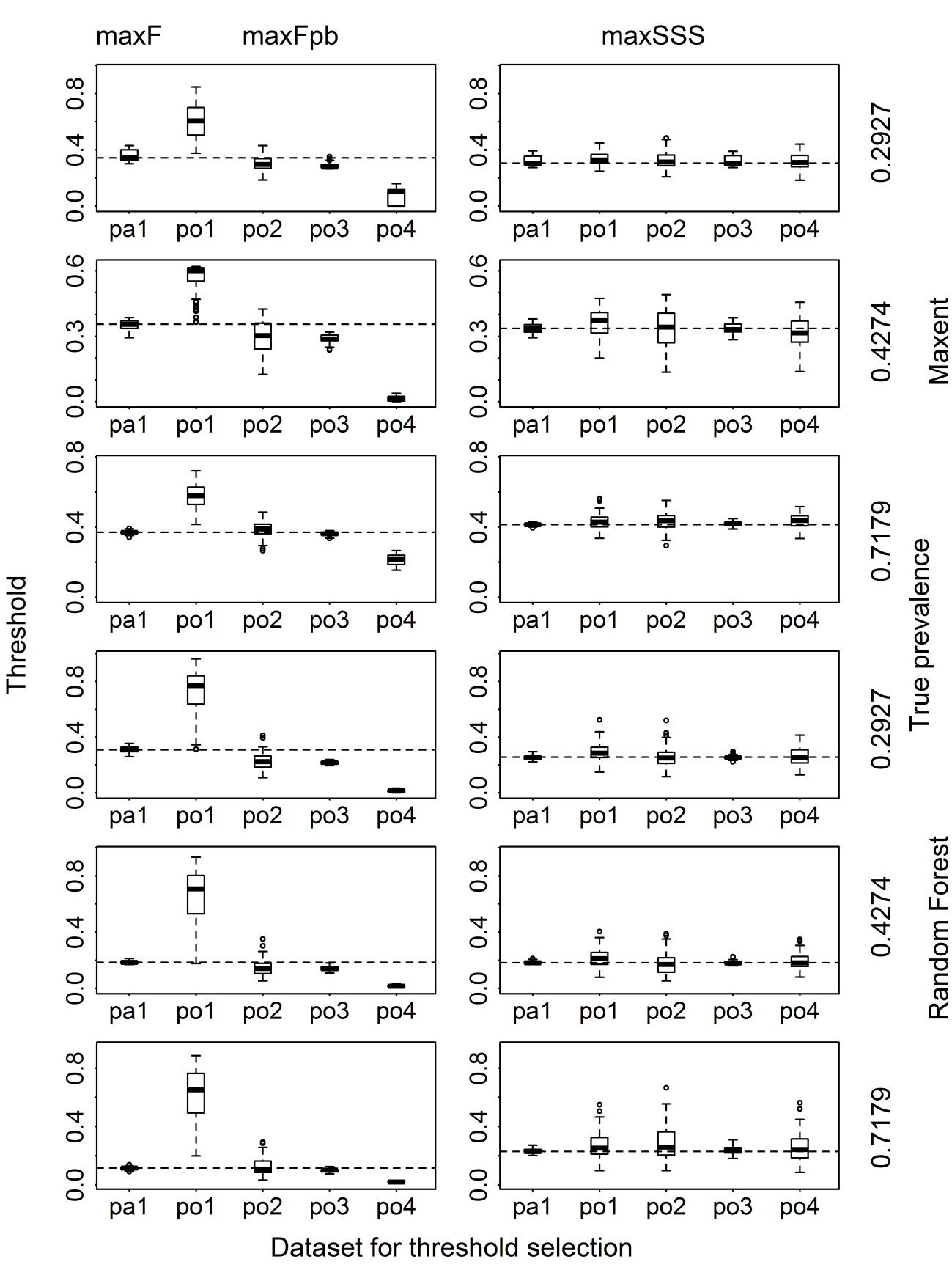


Fig. S3-2 Thresholds selected using maxF with presence/absence dataset (pa1), using maxF_pb_ with four presence-only datasets (po1, po2, po3 and po4) and using maxSSS with all the five datasets for Maxent and Random Forest models for three virtual species with three levels of prevalence (0.2927, 0.4274 and 0.7179). The dashed lines correspond to the median thresholds selected using maxF and maxSSS with pa1.


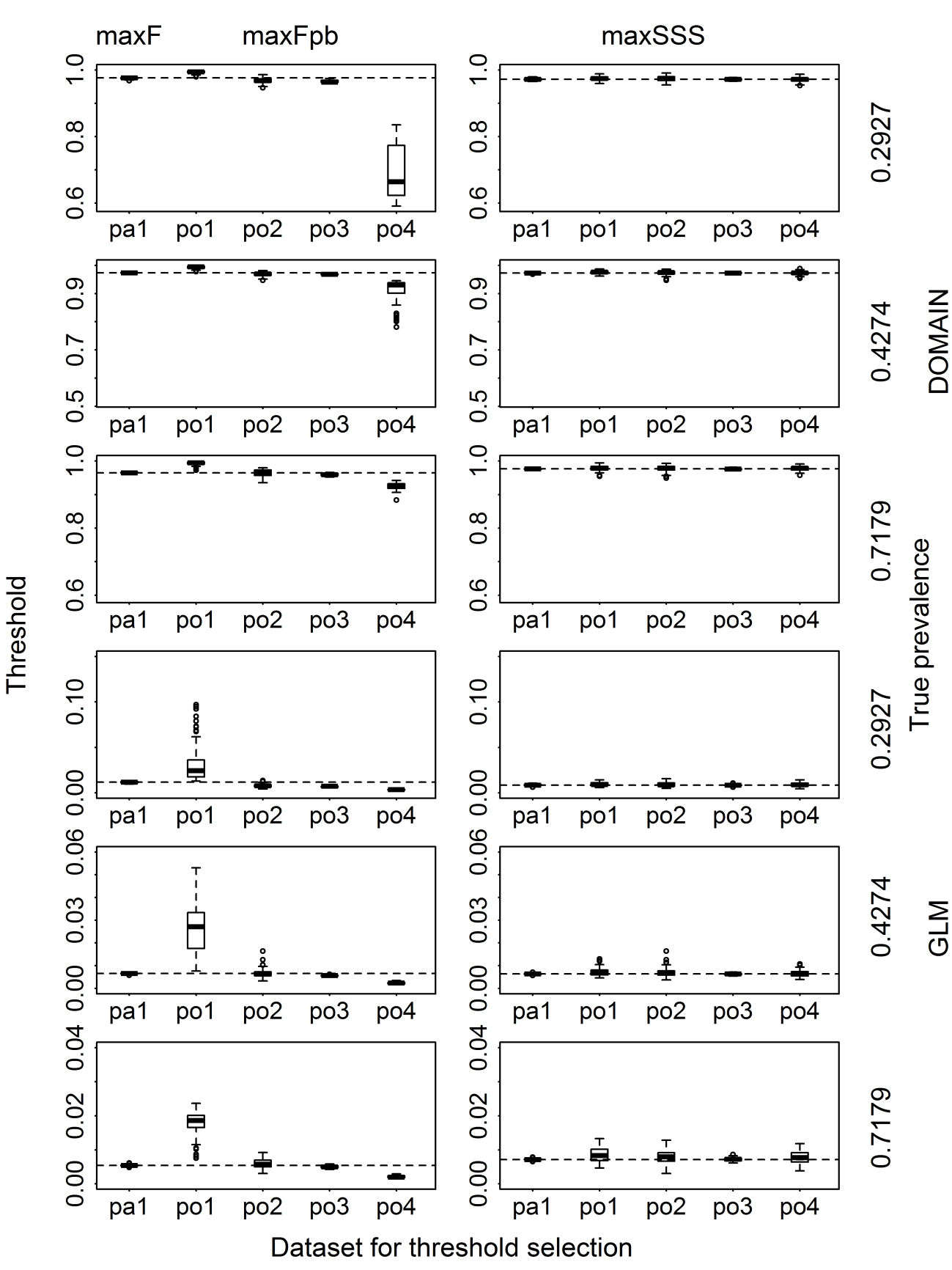


Fig. S3-3 Thresholds selected using maxF with presence/absence dataset (pa1), using maxF_pb_ with four presence-only datasets (po1, po2, po3 and po4) and using maxSSS with all the five datasets for DOMAIN and GLM models for three virtual species with three levels of prevalence (0.2927, 0.4274 and 0.7179). The dashed lines correspond to the median thresholds selected using maxF and maxSSS with pa1.


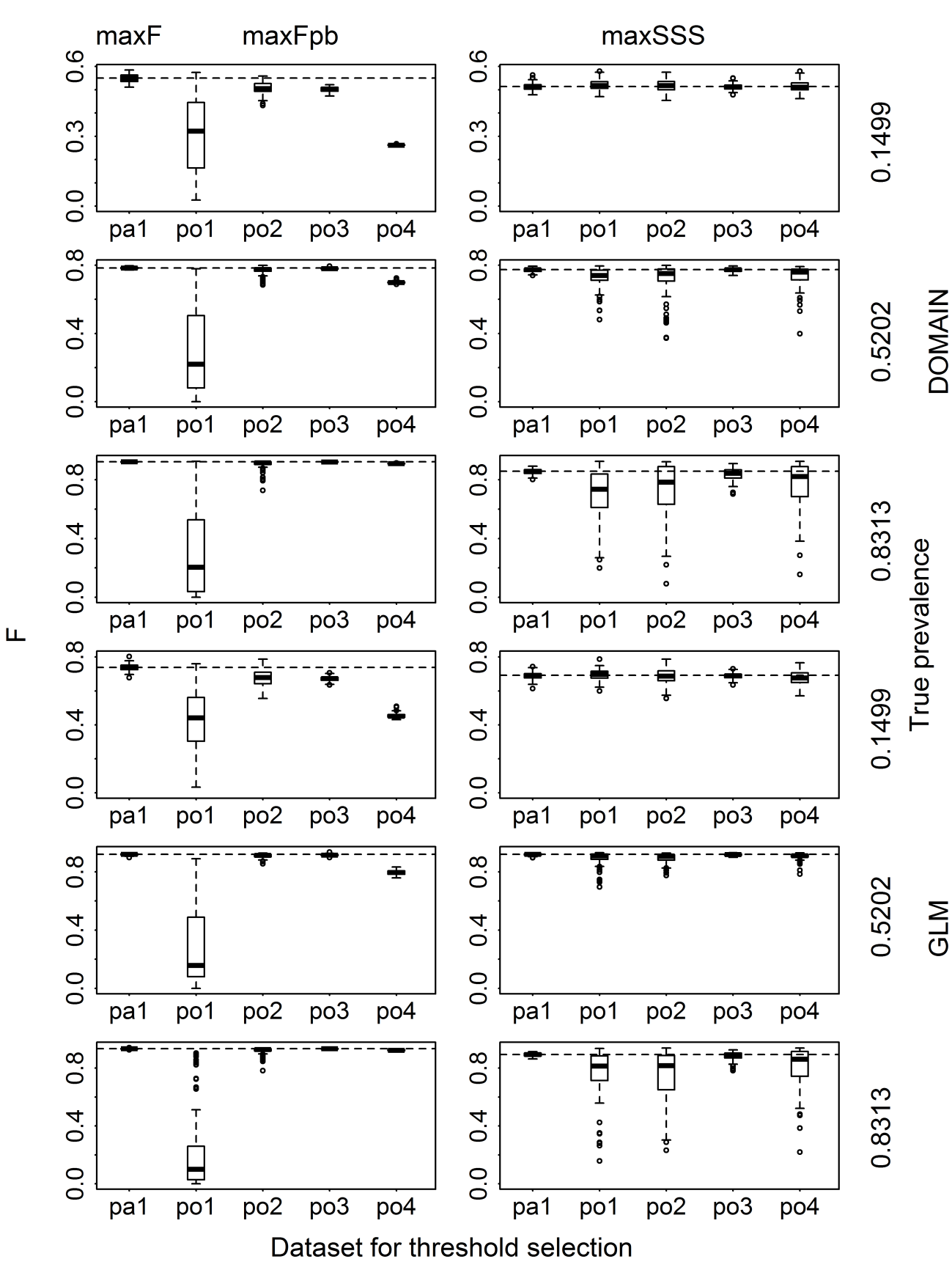


Fig. S3-4 F measures calculated for the results transformed with the thresholds selected using maxF with presence/absence dataset (pa1), using maxF_pb_ with four presence-only datasets (po1, po2, po3 and po4) and using maxSSS with all the five datasets for DOMAIN and GLM models for three virtual species with three levels of prevalence (0.1499, 0.5202 and 0.8313). The dashed lines correspond to the median F of those using maxF and maxSSS with pa1.


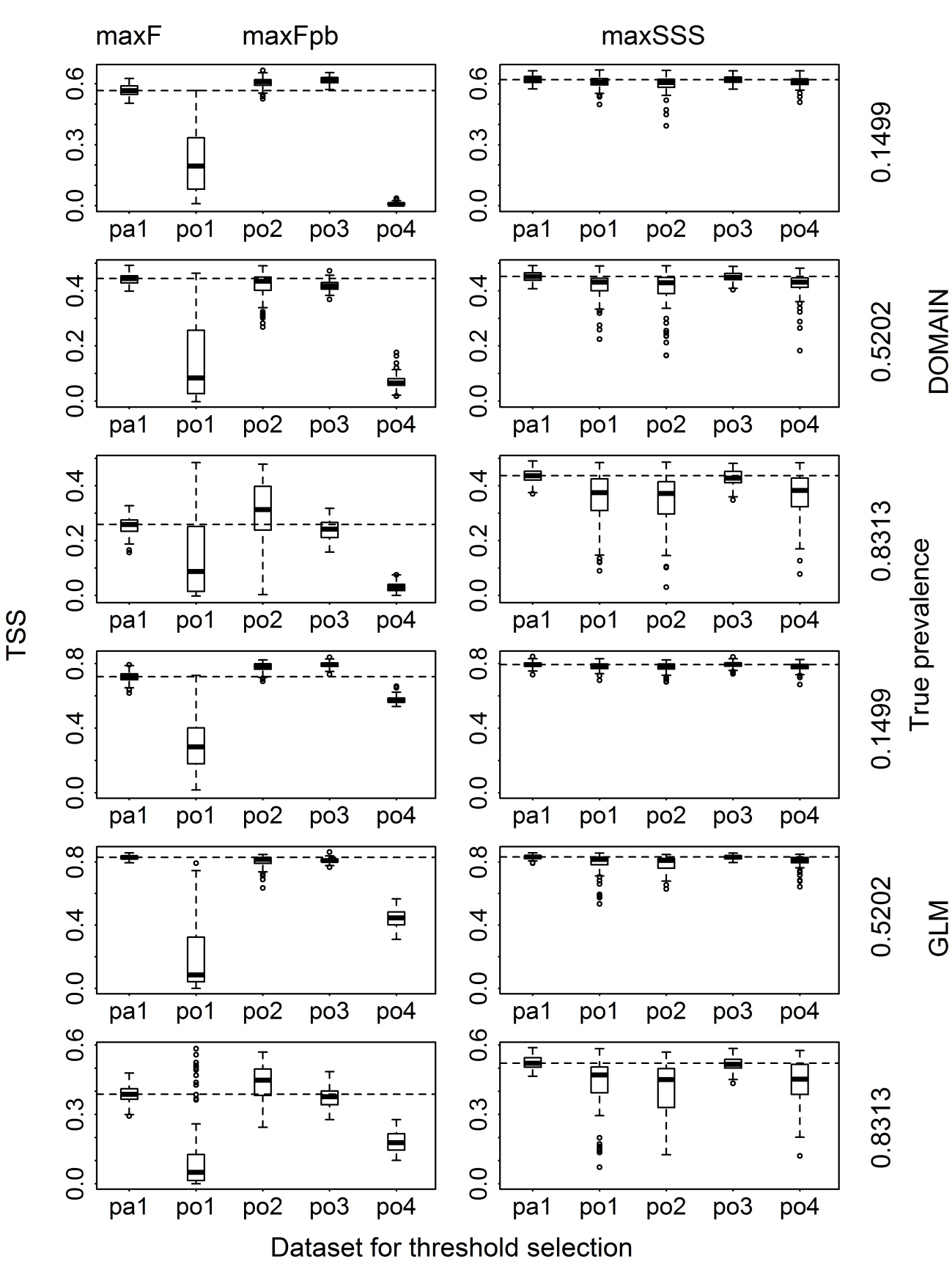


Fig. S3-5 True skill statistic (TSS) calculated for the results transformed with the thresholds selected using maxF with presence/absence dataset (pa1), using maxF_pb_ with four presence-only datasets (po1, po2, po3 and po4) and using maxSSS with all the five datasets for DOMAIN and GLM models for three virtual species with three levels of prevalence (0.1499, 0.5202 and 0.8313). The dashed lines correspond to the median TSS of those using maxF and maxSSS with pa1.


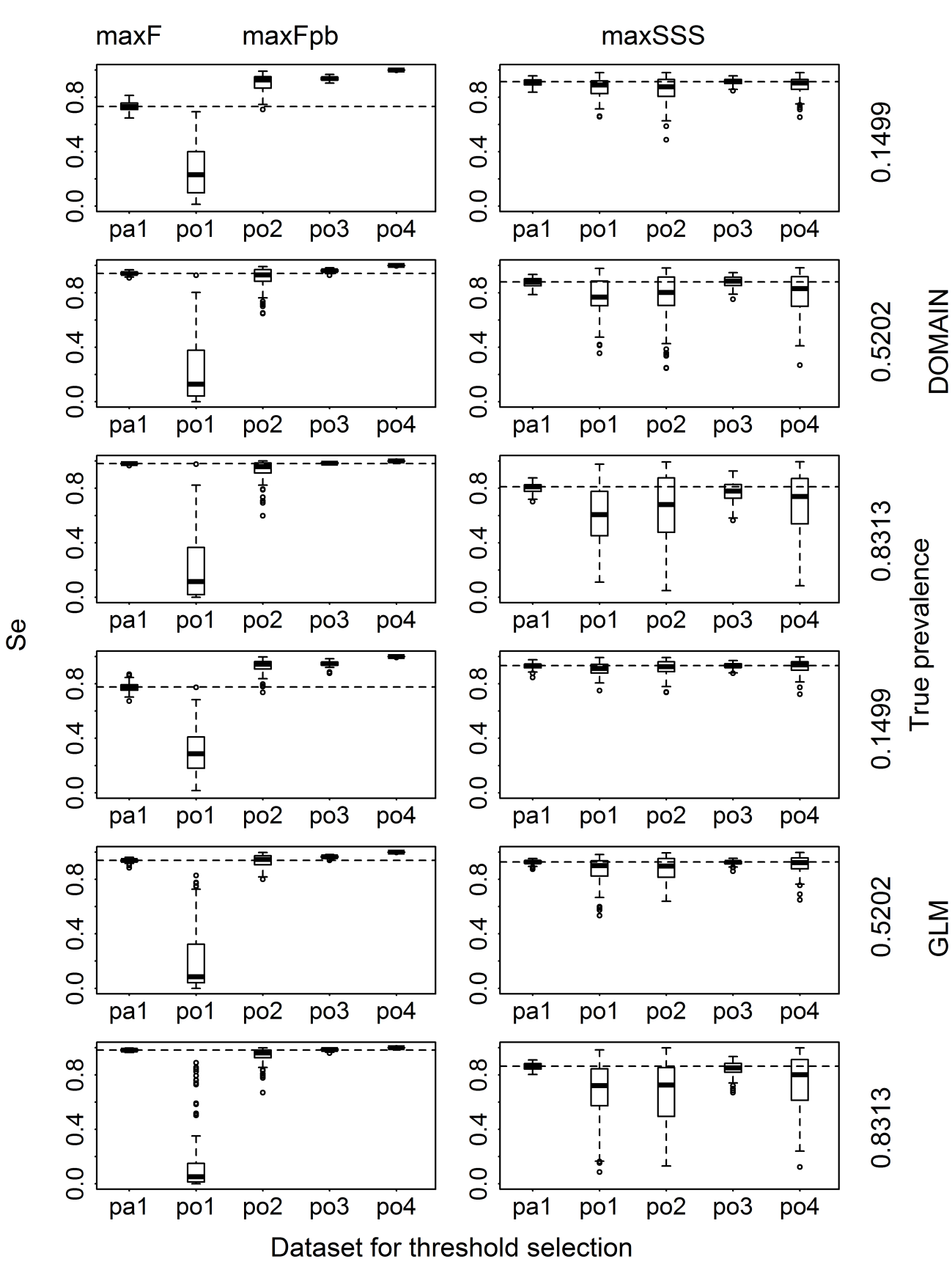


Fig. S3-6 Sensitivity (Se) calculated for the results transformed with the thresholds selected using maxF with presence/absence dataset (pa1), using maxF_pb_ with four presence-only datasets (po1, po2, po3 and po4) and using maxSSS with all the five datasets for DOMAIN and GLM models for three virtual species with three levels of prevalence (0.1499, 0.5202 and 0.8313). The dashed lines correspond to the median Se of those using maxF and maxSSS with pa1.


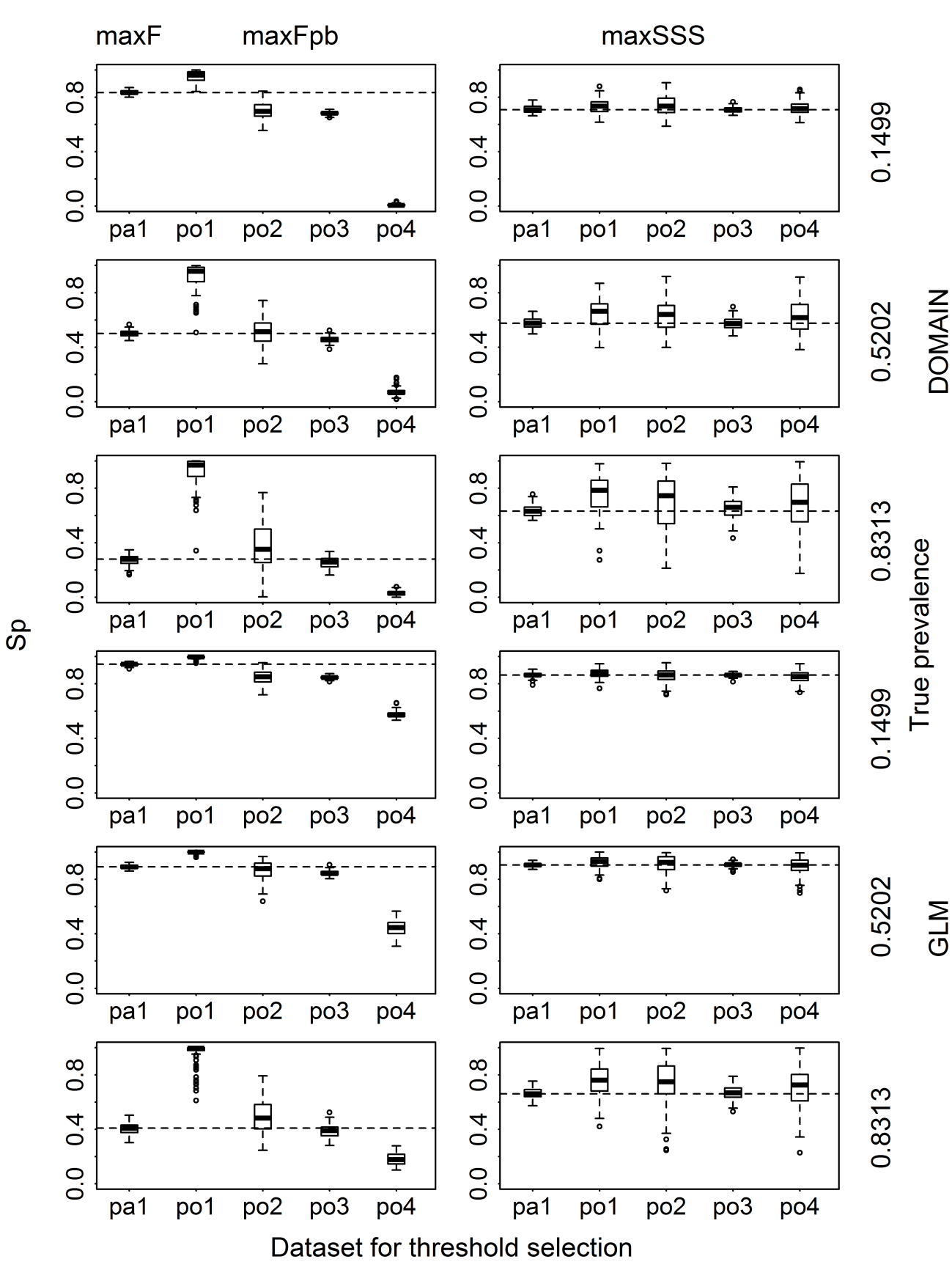


Fig. S3-7 Specificity (Sp) calculated for the results transformed with the thresholds selected using maxF with presence/absence dataset (pa1), using maxF_pb_ with four presence-only datasets (po1, po2, po3 and po4) and using maxSSS with all the five datasets for DOMAIN and GLM models for three virtual species with three levels of prevalence (0.1499, 0.5202 and 0.8313). The dashed lines correspond to the median Sp of those using maxF and maxSSS with pa1.


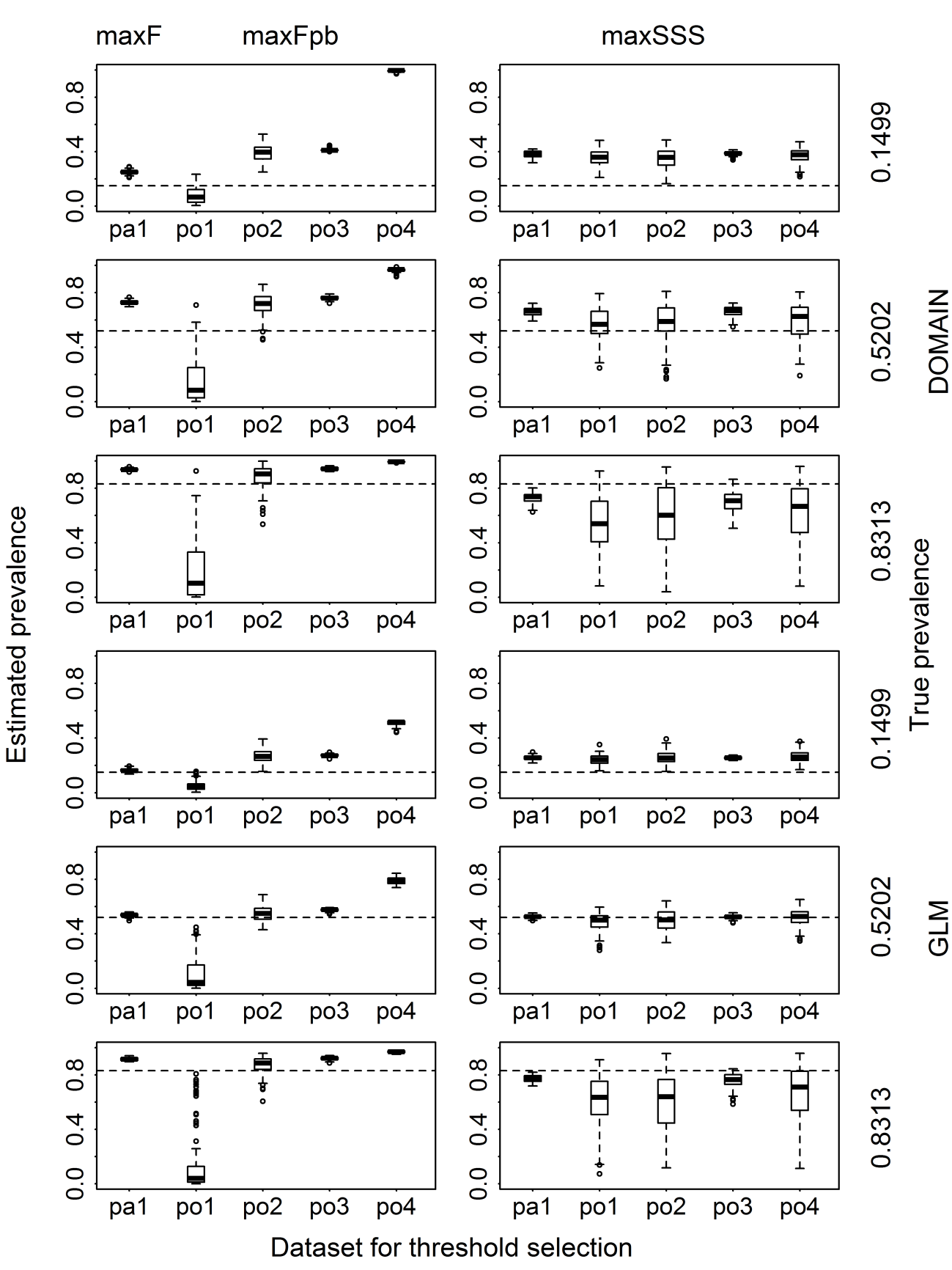


Fig. S3-8 Estimated prevalence from the results transformed with the thresholds selected using maxF with presence/absence dataset (pa1), using maxF_pb_ with four presence-only datasets (po1, po2, po3 and po4) and using maxSSS with all the five datasets for DOMAIN and GLM models for three virtual species with three levels of prevalence (0.1499, 0.5202 and 0.8313). The dashed lines correspond to the true prevalence.


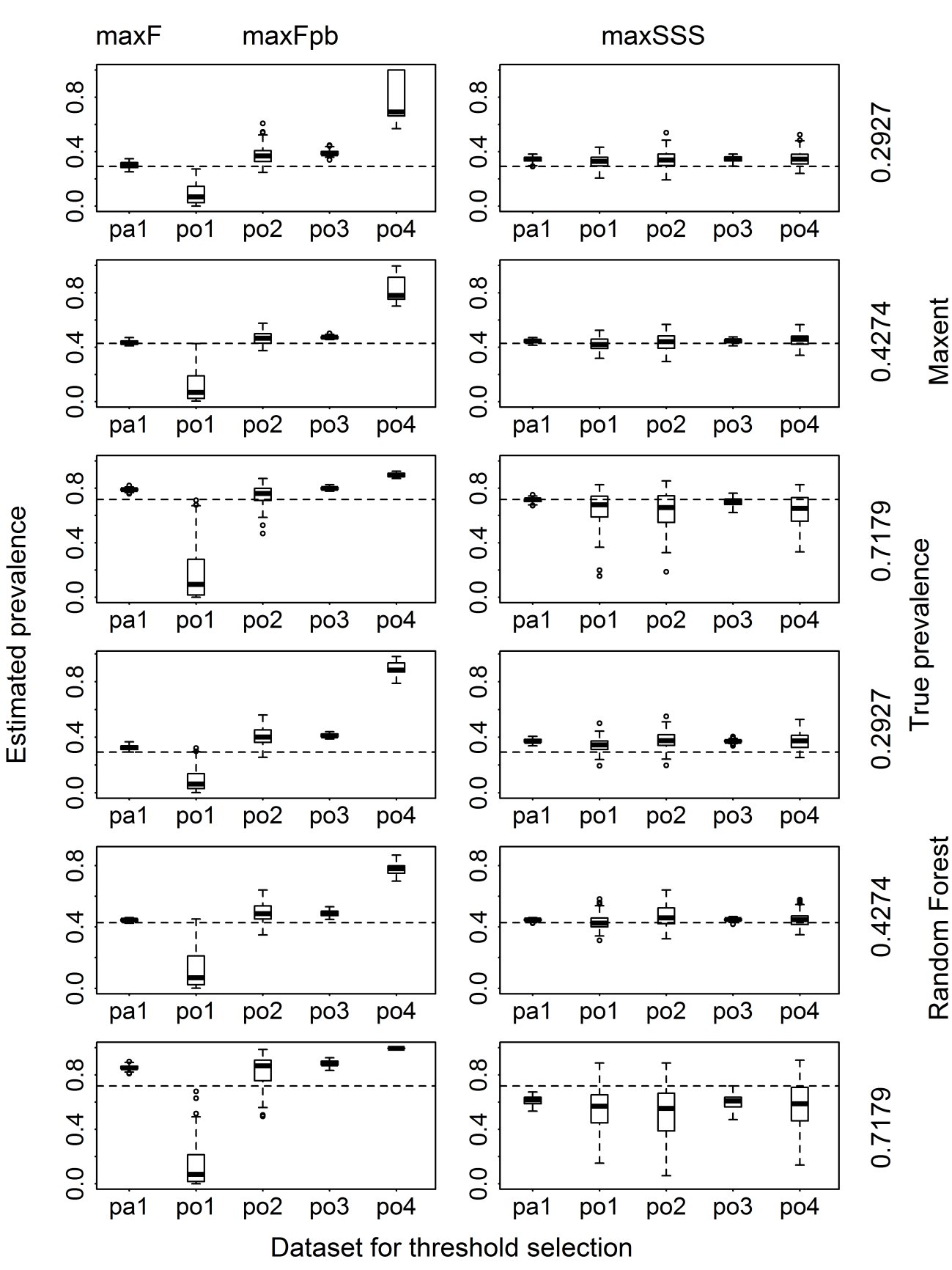


Fig. S3-9 Estimated prevalence from the results transformed with the thresholds selected using maxF with presence/absence dataset (pa1), using maxF_pb_ with four presence-only datasets (po1, po2, po3 and po4) and using maxSSS with all the five datasets for Maxent and Random Forest models for three virtual species with three levels of prevalence (0.2927, 0.4274 and 0.7179). The dashed lines correspond to the true prevalence.


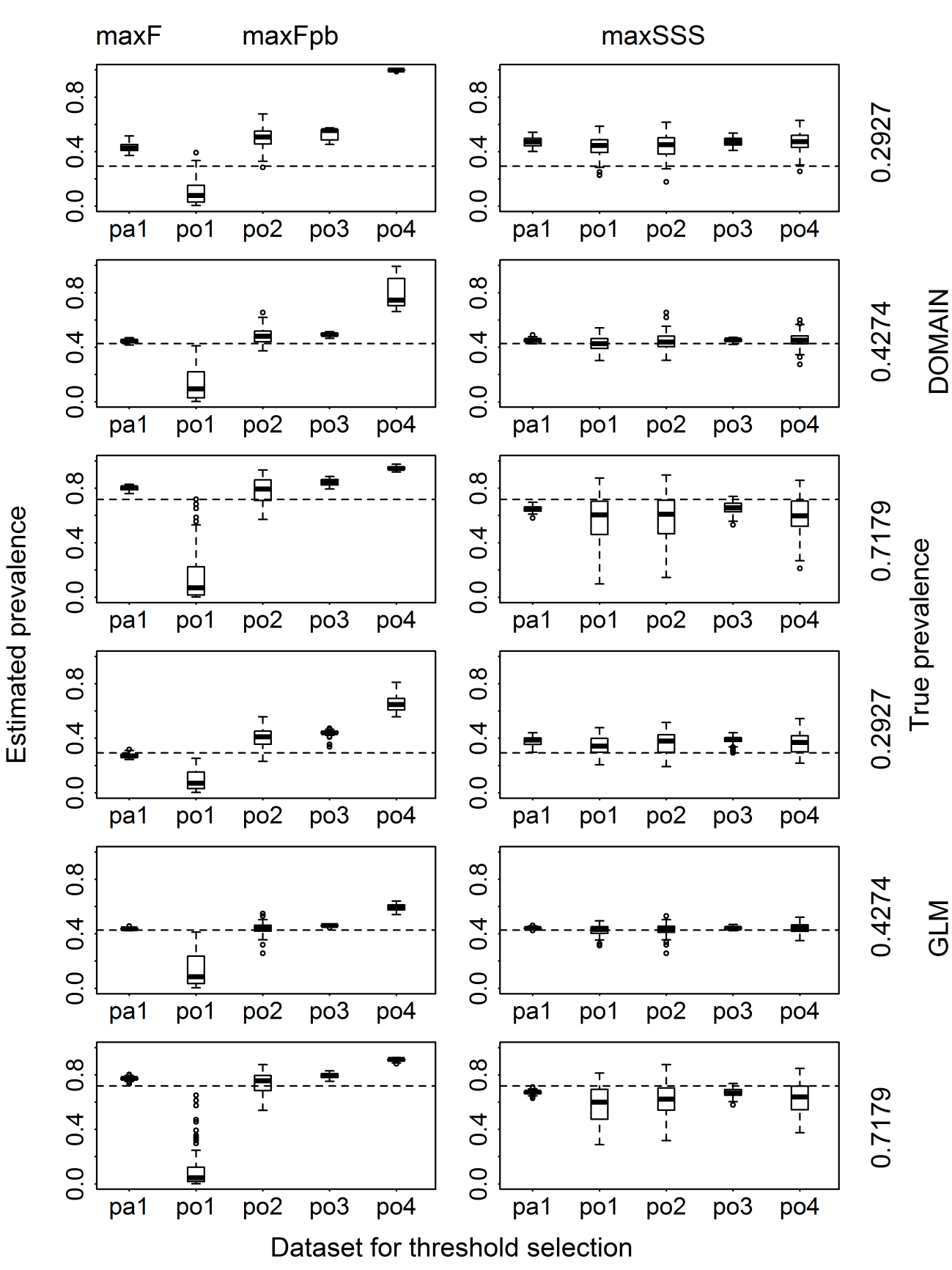


Fig. S3-10 Estimated prevalence from the results transformed with the thresholds selected using maxF with presence/absence dataset (pa1), using maxF_pb_ with four presence-only datasets (po1, po2, po3 and po4) and using maxSSS with all the five datasets for DOMAIN and GLM models for three virtual species with three levels of prevalence (0.2927, 0.4274 and 0.7179). The dashed lines correspond to the true prevalence.
